# Tailored risk assessment and forecasting in intermittent claudication

**DOI:** 10.1093/bjsopen/zrad166

**Published:** 2024-02-27

**Authors:** Bharadhwaj Ravindhran, Jonathon Prosser, Arthur Lim, Bhupesh Mishra, Ross Lathan, Louise H Hitchman, George E Smith, Daniel Carradice, Ian C Chetter, Dhaval Thakker, Sean Pymer

**Affiliations:** Academic Vascular Surgical Unit, Allam Diabetes Centre, Hull Royal Infirmary, Hull, UK; Department of Health Sciences, University of York, York, UK; Academic Vascular Surgical Unit, Allam Diabetes Centre, Hull Royal Infirmary, Hull, UK; Academic Vascular Surgical Unit, Allam Diabetes Centre, Hull Royal Infirmary, Hull, UK; School of Computer Science, University of Hull, Hull, UK; Academic Vascular Surgical Unit, Allam Diabetes Centre, Hull Royal Infirmary, Hull, UK; Academic Vascular Surgical Unit, Allam Diabetes Centre, Hull Royal Infirmary, Hull, UK; Academic Vascular Surgical Unit, Allam Diabetes Centre, Hull Royal Infirmary, Hull, UK; Academic Vascular Surgical Unit, Allam Diabetes Centre, Hull Royal Infirmary, Hull, UK; Academic Vascular Surgical Unit, Allam Diabetes Centre, Hull Royal Infirmary, Hull, UK; School of Computer Science, University of Hull, Hull, UK; Academic Vascular Surgical Unit, Allam Diabetes Centre, Hull Royal Infirmary, Hull, UK

## Abstract

**Background:**

Guidelines recommend cardiovascular risk reduction and supervised exercise therapy as the first line of treatment in intermittent claudication, but implementation challenges and poor patient compliance lead to significant variation in management and therefore outcomes. The development of a precise risk stratification tool is proposed through a machine-learning algorithm that aims to provide personalized outcome predictions for different management strategies.

**Methods:**

Feature selection was performed using the least absolute shrinkage and selection operator method. The model was developed using a bootstrapped sample based on patients with intermittent claudication from a vascular centre to predict chronic limb-threatening ischaemia, two or more revascularization procedures, major adverse cardiovascular events, and major adverse limb events. Algorithm performance was evaluated using the area under the receiver operating characteristic curve. Calibration curves were generated to assess the consistency between predicted and actual outcomes. Decision curve analysis was employed to evaluate the clinical utility. Validation was performed using a similar dataset.

**Results:**

The bootstrapped sample of 10 000 patients was based on 255 patients. The model was validated using a similar sample of 254 patients. The area under the receiver operating characteristic curves for risk of progression to chronic limb-threatening ischaemia at 2 years (0.892), risk of progression to chronic limb-threatening ischaemia at 5 years (0.866), likelihood of major adverse cardiovascular events within 5 years (0.836), likelihood of major adverse limb events within 5 years (0.891), and likelihood of two or more revascularization procedures within 5 years (0.896) demonstrated excellent discrimination. Calibration curves demonstrated good consistency between predicted and actual outcomes and decision curve analysis confirmed clinical utility. Logistic regression yielded slightly lower area under the receiver operating characteristic curves for these outcomes compared with the least absolute shrinkage and selection operator algorithm (0.728, 0.717, 0.746, 0.756, and 0.733 respectively). External calibration curve and decision curve analysis confirmed the reliability and clinical utility of the model, surpassing traditional logistic regression.

**Conclusion:**

The machine-learning algorithm successfully predicts outcomes for patients with intermittent claudication across various initial treatment strategies, offering potential for improved risk stratification and patient outcomes.

## Teaser

The study proposes a machine-learning algorithm for precise risk stratification in patients with intermittent claudication, aiming to provide personalized outcome predictions for different management strategies. The algorithm was developed using a bootstrapped sample of 10 000 patients and evaluated using the area under the receiver operating characteristic curve, calibration curves, and decision curve analysis. The results showed excellent discrimination and consistency between predicted and actual outcomes, surpassing traditional logistic regression, suggesting that the algorithm can successfully predict outcomes for intermittent claudication patients across various initial treatment strategies, potentially improving risk stratification and patient outcomes.

## Introduction

Patients with intermittent claudication (IC) are at a heightened risk of chronic limb-threatening ischaemia (CLTI), major adverse cardiovascular events (MACE), major adverse limb events (MALE)^1–3^, and therefore death^4^. The European Society for Vascular Surgery^3^ and the National Institute for Health and Care Excellence^5^ both recommend first-line treatment strategies that encompass cardiovascular risk reduction and supervised exercise therapy (SET). However, implementation challenges, such as the non-availability of SET, compounded by poor patient uptake, adherence, and completion rates, mean there is significant variation in the management of these patients. This includes the adoption of a percutaneous transluminal angioplasty (PTA)-first strategy, despite poor patient compliance with smoking cessation and secondary prevention^6,7^. This variation underscores the pressing need for accurate risk prediction models for different management strategies for patients with IC. Machine learning (ML) can be used to develop such models.

Current ML-based predictive models for peripheral arterial disease (PAD) focus primarily on baseline characteristics, such as age and smoking status, and often overlook the non-linear interaction between these factors and other critical factors, such as compliance with best medical therapy (BMT), SET, smoking cessation, and lifestyle modification^8–14^. Consideration of these factors is essential for accurately predicting final outcomes. These models and nomograms can therefore predict death, albeit grossly, but their translation into real-time precise risk forecasting based on compliance and management strategies is limited. This also limits their use pragmatically, as they are unable to assist in the determination of the optimal management strategy for each patient. To address this, the development of a precise risk stratification model is proposed through an ML algorithm that aims to provide personalized outcome predictions for different management strategies, accounting for factors such as compliance with BMT and smoking cessation. This approach aims not only to enhance the prognosis and management of IC but also to address 6 of the 10 critical questions posed by the James Lind Alliance Priority Setting Partnership in PAD. These questions pertain to earlier/better diagnosis of IC and its long-term impact, education of healthcare professionals and patients to improve the understanding of the consequences of IC, slowing down symptom progression in IC, and preventing and reducing the overall cardiovascular risk in PAD^15^.

## Methods

### Study design

A bootstrapped sample based on an anonymized retrospective data set of patients with intermittent claudication from a tertiary vascular centre was utilized to develop an ML-based risk prediction model, which considered 27 baseline characteristics, compliance with BMT/smoking cessation, and treatment strategy, that is BMT (pharmacotherapy and exercise advice), SET, endovascular intervention (EI), and SET + EI (*Appendix S1*). These data were then validated in a similar data set consisting of patient data that was not used in model development.

### Patient characteristics in the data set

The data set consisted of patients with IC (Rutherford 1–3) who were referred to the vascular clinic from December 2014 to March 2018. Their clinical progression was followed from the time of presentation until the most recent follow-up, including baseline characteristics, compliance with BMT (lipid-lowering agents and antiplatelet agents), enrolment and completion of SET, and time to MALE, MACE, and CLTI. The data of patients who had CLTI at index presentation or had a recurrence of symptoms after previous revascularization were excluded from the training data set. The diagnosis of IC was made clinically and was further supported by a resting ankle brachial pressure index or toe brachial pressure index, duplex ultrasonography, or cross-sectional imaging if required. Patients who declined SET were discharged back to their general practitioner, received regular follow-up, or underwent a revascularization procedure, depending on disease progression.

### Interventions

#### Best medical therapy

All patients were initiated on BMT, which included antiplatelet therapy (aspirin and/or clopidogrel), smoking cessation advice and offer of support through the National Health Service smoking cessation programme, and nicotine replacement therapy. Additionally, risk-factor modification was implemented based on evidence-based care pathways, including goal-oriented management of diabetes, control of hypertension, and treatment of hypercholesterolaemia. Patients were also given advice leaflets on physical activity and exercise. Compliance with these measures was recorded through retrospective record review. Compliance was assessed based on the surgeon’s or clinician’s judgement of appropriate adherence during visits.

#### Endovascular interventions

An EI, including PTA and/or primary stenting, was performed by a consultant vascular radiologist, according to a predefined protocol, in accordance with the normal practice of the unit, in a dedicated vascular radiology suite.

#### Supervised exercise therapy

Patients underwent SET three times per week for 12 weeks, totalling 36 sessions. Any missed sessions were rescheduled and completed at the end of the 12-week programme^16^. The programme was supervised by an exercise physiologist, with assistance from undergraduate and postgraduate sports science students. Each SET session consisted of a circuit comprising six 2-min stations, with 2-min walking intervals in-between. Before the circuit, a warm-up was performed, followed by a cool down. The stations included step-ups, standing knee bends, sitting knee extensions, biceps curls, cycling, and heel raises. As a patient’s exercise tolerance improved, one additional station was added each week, starting from the seventh week. By the end of week 12, patients completed two full circuits. The duration of each session ranged from 30 to 60 min. Successful completion of SET was defined as the completion of 36 sessions. This circuit-based training programme was developed based on previous recommendations that emphasized the effectiveness of combining upper and lower limb ergometry, resistance exercise, and walking-based exercises to enhance muscle strength and cardiorespiratory fitness. These interventions have demonstrated a greater cardiorespiratory stimulus compared with walking alone^17–20^.

#### Combined therapy

Combined treatment was defined as undergoing an EI, according to the protocol described above, followed by the initiation of SET, 1 week after the procedure.

### Exclusion criteria

Patients with CLTI (Rutherford 4 and above) at their initial presentation or those who experienced a recurrence of symptoms after previous revascularization were excluded from the study. Additionally, patients who were referred for SET but deemed unsuitable due to contraindications or significant co-morbidities were also excluded.

### Outcome measures

The outcomes of interest included the risk of progression to CLTI at 2 years, the risk of progression to CLTI at 5 years, the likelihood of major adverse cardiovascular events within 5 years, the likelihood of major adverse limb events within 5 years, and the likelihood of two or more revascularization procedures (on the index side) within 5 years, starting from the date of the clinic visit when the intervention was offered. CLTI was defined as ischaemic rest pain lasting for 2 or more weeks, non-healing wounds, or gangrene that was attributable to objectively proven arterial occlusive disease. A MACE was defined as non-fatal stroke, non-fatal myocardial infarction, or cardiovascular death^21^. A MALE was defined as acute limb ischaemia, untreated loss of patency, or major lower limb amputation^22^.

### Feature selection and machine-learning analysis

The model was designed to utilize routinely collected data available at the time of presentation in the clinic (*Appendix S1*), thereby ensuring its relevance to everyday vascular surgical practice. Feature selection was performed using the least absolute shrinkage and selection operator (LASSO) method, which assigned weights to each clinical parameter to accurately predict outcomes for each initial management strategy^23^. The LASSO method was applied to these variables to select the most relevant features for predicting the outcome. The form of the model equation is a linear combination of the selected features, with the coefficients determined by the LASSO method. The model was rigorously evaluated using 10 times repeated 5-fold nested cross-validation with a fixed seed to enhance robustness^24^. The first data set was split into five training (70%) and internal validation (30%) sets. The hyperparameters of the ML models were optimized on these training and validation sets via a grid search before final evaluation on the validation set. In this paper, the focus during model training was primarily on capturing the main effects of the variables. However, the potential presence of interaction effects between variables was also taken into account using the LASSO package in Stata 18. Missing data in the training and validation sets were compensated for using a multiple imputation by chained equations (MICE) framework^25^, which allows for the imputation of missing values using multiple iterations. Within the MICE framework, the predictive mean matching method was specifically utilized. In this study, the missing data were less than 5%, primarily observed in variables such as serum albumin, compliance with antiplatelet medication, and self-reported claudication distance. The predictive mean matching method was applied to impute the missing values for these variables. This technique selects observed values from similar individuals to impute the missing values, ensuring that the imputed values are plausible and reflect the underlying distribution of the variable.

Outcome class imbalance, a common issue in ML, refers to an unequal class distribution in the training data set. This can cause model bias towards the majority class, affecting its generalization ability. The imbalance was addressed in this case using random oversampling^26^. This technique involved identifying the minority class (MACE), which had fewer instances, and then randomly duplicating instances from this class. The selection process for duplication was random, ensuring each instance in the minority class had an equal chance of being selected. This duplication was repeated until the number of instances in the minority class was approximately equal to that in the majority class, thereby balancing the class distribution. The oversampled data set, which included both the original and duplicated instances, was then used for training the ML model.

The final model was then validated in an external data set of patients that was not used for training, thereby accounting for overfitting^27^. Model performance was evaluated using the area under the receiver operating characteristic (AUROC) for each outcome. The confidence intervals for the AUROC were calculated using the binomial exact method, which is a non-parametric approach to construct confidence intervals for a proportion in a statistical population. Calibration curves were generated to assess the consistency between predicted and actual outcomes. Decision curve analysis (DCA) was performed to evaluate the clinical usefulness of the model by quantifying the net benefits at different threshold probabilities^28^.

This study adhered to the transparent reporting of a multivariable prediction model for individual prognosis or diagnosis (‘TRIPOD’) guidelines^29^.

### Sample size calculation

It is generally recommended to have a minimum of 100 events and 100 non-events when conducting a validation study to assess the performance of a model using new data from the same or a different population. However, the question of determining an adequate sample size in ML remains an ongoing challenge. It is widely recognized that simply having a large sample size alone is not sufficient for reliable hypothesis testing^30^. It has recently been reported that the suitability of a sample size for ML-based prediction is contingent upon ML accuracy greater than 80%^31^. Therefore, based on the available data and considering the established guidelines, a sample size of some 250 patients in the training and validation sets is well suited for evaluating the performance of this proof-of-concept ML algorithm. However, it is important to note that this assessment of sample adequacy is based on the observed performance of the ML model rather than an a-priori calculation.

### Statistical analysis

Statistical analysis was performed using Stata 18 (StataCorp LLC, College Station, TX, USA; 2023), SPSS^®^ (IBM, Armonk, NY, USA; version 26.0; 2019), and MedCalc (MedCalc Software Ltd, Ostend, Belgium; https://www.medcalc.org; version 19.2.6; 2020). The efficacy of each model was compared using receiver operating characteristic (ROC) curve analysis, with the AUROC calculated using predicted values. A DeLong test was used to compare ROC curves^32^. Statistical significance was determined by assigning a two-sided significance level of α = 0.050, unless otherwise specified.

## Results

A bootstrapped sample of 10 000 based on a training data set of 255 patients was utilised. There were a further 254 patients included in the testing data set. Patient characteristics of the two datasets are shown in Table S1. In the training set, the incidence of CLTI within 2 years was 27.4% (70 patients), the incidence of CLTI within 5 years was 45.9% (117 patients), the requirement for two or more revascularization procedures within 5 years was 50.6% (129 patients), MALE was 41.6% (106 patients), and MACE was 41.6% (106 patients). Similarly, in the testing set, the incidence of CLTI within 2 years was 29.1% (74 patients), the incidence of CLTI within 5 years was 48.8% (124 patients), the requirement for two or more revascularization procedures within 5 years was 54.3% (138 patients), the incidence of MALE within 5 years was 52.4% (133 patients), and MACE was 49.2% (125 patients).

The developed model assigned the highest coefficients to factors such as hypertension, ischaemic heart disease, BMI, atrial fibrillation, self-reported claudication distance, non-completion of SET (that is a treatment strategy of BMT or EI alone), duration of smoking, and the presence of bilateral iliac/crural vessel disease. The model demonstrated its comprehensive analytical capability by dynamically assigning weights to each of the 30 variables, including baseline characteristics, compliance data, and adopted treatment strategy for each outcome.

**Fig. 1:**
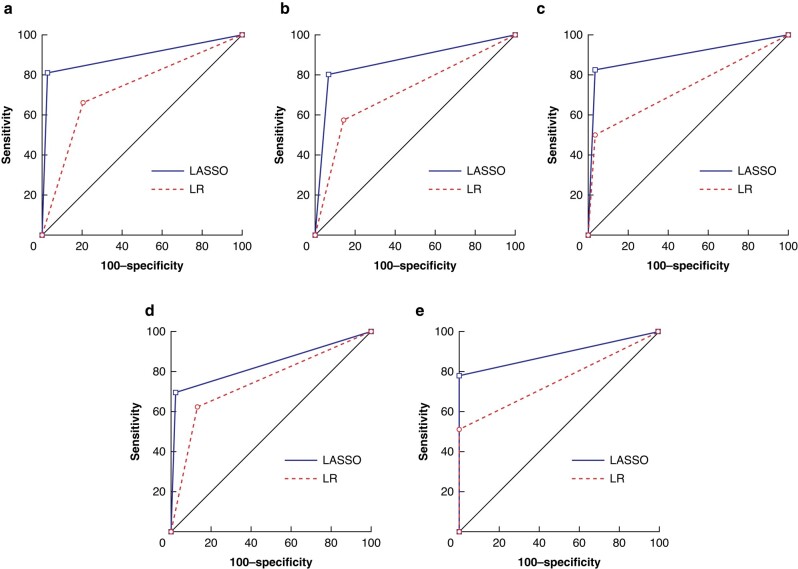
Area under the receiver operating characteristic of the least absolute shrinkage and selection operator algorithm *versus* logistic regression algorithm in the likelihood of prediction of adverse outcomes **a** Risk of progression to chronic limb-threatening ischaemia at 2 years. **b** Risk of progression to chronic limb-threatening ischaemia at 5 years. **c** Likelihood of two or more revascularization procedures within 5 years. **d** Likelihood of major adverse cardiovascular events within 5 years. **e** Likelihood of major adverse limb events within 5 years. LASSO, least absolute shrinkage and selection operator; LR, logistic regression.

The LASSO algorithm demonstrated an excellent discriminatory capacity for various outcomes based on the initial management strategy. The comparative predictive abilities for the ML algorithm and logistic regression are shown in *Table 1* and *Fig. 1*.

**Table 1:** Comparison of algorithm performance in the likelihood of prediction of adverse outcomes

Outcome	Predictive ability based on initial management strategy	
	Machine-learning algorithm AUROC curve (95% c.i.), s.e.	Logistic regression AUROC curve (95% c.i.), s.e.
Risk of progression to CLTI at 2 years	0.892 (0.847,0.927), 0.024	0.728 (0.669,0.782), 0.032
Risk of progression to CLTI at 5 years	0.866 (0.818,0.906), 0.021	0.717 (0.657,0.771), 0.0272
Likelihood of MACE within 5 years	0.836 (0.785,0.880), 0.217	0.746 (0.688,0.798), 0.267
Likelihood of MALE within 5 years	0.891 (0.846,0.927), 0.018	0.756 (0.698,0.807), 0.022
Likelihood of two or more revascularization procedures within 5 years	0.896 (0.852,0.931), 0.018	0.733 (0.674,0.786), 0.023

AUROC, area under the receiver operating characteristic; CLTI, chronic limb-threatening ischaemia; MACE, major adverse cardiovascular events; MALE, major adverse limb events.

**Fig. 2:**
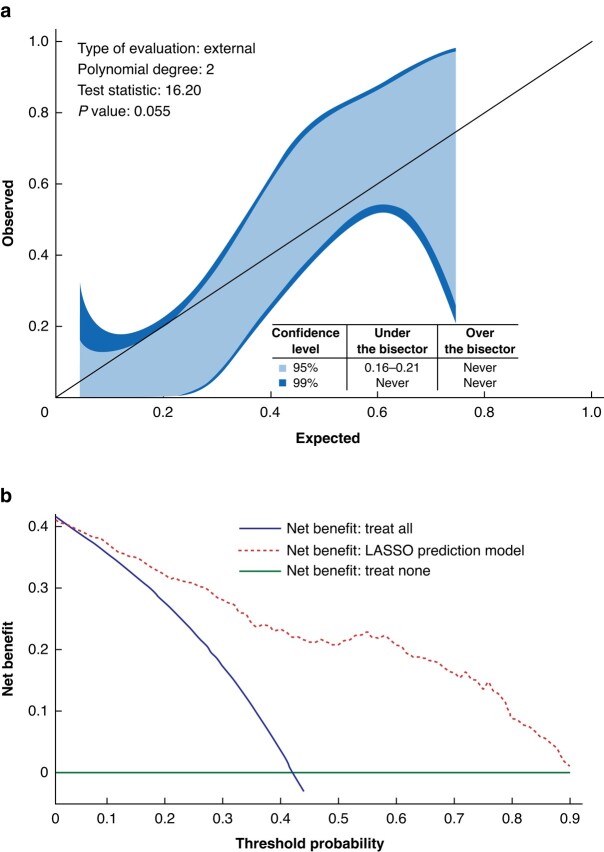
External calibration curve and decision curve analysis curve of the least absolute shrinkage and selection operator (LASSO) algorithm **a** Calibration curve, demonstrating good consistency between the predicted and actual outcomes. **b** The decision curve analysis shows that the LASSO model has a better overall clinical utility, demonstrating a predictive accuracy greater than 45%.

The reliability of the model was further confirmed by an external calibration curve, which showed a high degree of consistency between the predicted and actual outcomes of interest (test statistic = 16.2; *P* = 0.055) (*Fig. 2a*). This performance was consistent, regardless of the initial treatment strategy. DCA validated the clinical usefulness of the model, showing a significant advantage of greater than 45% by efficiently balancing potential benefits and harms. DCA was conducted to assess the clinical usefulness of the model, specifically comparing it with the ‘treat all’ and ‘treat none’ strategies. The results of the DCA demonstrated a significant advantage of greater than 45% when using the model, indicating that it efficiently balances the potential benefits and harms associated with the decision-making process. This suggests that the model outperforms both the ‘treat all’ and ‘treat none’ strategies in terms of clinical utility. By considering the net benefit of the model, DCA provided valuable insights into the potential benefits of using the model for decision-making, further supporting its practical application in clinical settings (*Fig. 2b*).

## Discussion

This study underscores the significant potential of ML models compared with traditional logistic regression-based models in accurately predicting adverse outcomes in patients with IC. The developed model goes beyond considering baseline characteristics and incorporates compliance data to predict outcomes for different treatment strategies. The model exhibited an excellent discriminatory capacity, as evidenced by high AUROC values, and demonstrated clinical utility, as indicated by DCA results. Notably, this study stands out as one of the first to incorporate treatment strategies into outcome prediction, whereas the existing literature primarily focuses on baseline characteristics alone. By considering the impact of treatment strategies, this model provides a more comprehensive and nuanced approach to outcome prediction in IC patients. Furthermore, this study distinguishes itself due to the conduct of external validation, which adds further credibility to the model’s performance and generalizability. The inclusion of external validation enhances the robustness of the findings and strengthens the applicability of the model in real-world clinical settings.

Accurate risk prediction models are crucial for the management of IC, as they enable personalized treatment strategies. Current ML models primarily focus on the diagnosis of PAD. Whilst there are a few existing ML-based prediction models, it is important to note that they are not specific to IC but rather encompass the entire spectrum of PAD^8–14^. In contrast, the present study stands out as the first to examine the interaction between treatments offered and compliance, which has the potential to significantly impact clinical practice. By identifying high-risk patients at their initial presentation to the vascular clinic, these findings have the potential to bring about practice-changing implications. Whilst there have been two studies that have utilized large data sets to predict adverse outcomes, such as MALE and MACE, they predominantly rely on baseline clinical characteristics as predictors^14,33^. However, an important factor that significantly influences outcomes, namely compliance with medications and treatment strategies offered, is often overlooked in these models. Therefore, there is a critical gap in the existing literature, in which the incorporation of compliance data and treatment strategies in ML models is lacking. Addressing this gap is crucial for developing more comprehensive and accurate predictive models for adverse outcomes in PAD patients^8–11^.

This study serves as a preliminary step towards addressing this by proposing an ML model that provides personalized outcome predictions for different levels of compliance and management strategies. The model can also identify patients who are high risk, prompting timely and effective care. For example, if the model predicts that a patient is likely to progress to CLTI, regardless of the treatment approach or compliance, they can be identified as high risk. The model’s projections can be utilized to motivate patients to adhere to their treatment plans, showcasing the potential for real-time risk reduction during their initial visit to the vascular clinic.

The ML model in the present study utilizes readily available variables at the first patient contact in a vascular clinic, increasing its practicality and applicability. The model’s robustness was validated in an external data set spanning over a decade, encompassing significant temporal management changes. Despite these changes, the model maintained its predictive accuracy, demonstrating resilience and adaptability to evolving clinical practices. This validation enhances the credibility of the model and supports its potential for widespread application. In the context of clinical decision-making, the balance, as confirmed by DCA, is crucial, as it helps healthcare professionals weigh the potential positive outcomes of a treatment or intervention against its possible negative effects. The model’s ability to account for this balance enhances its utility in a clinical setting. It provides a more comprehensive view of the potential outcomes, allowing for more informed and effective decision-making. This is particularly important in scenarios where the benefits and harms are closely matched and even a slight shift in balance could significantly impact a patient’s health outcome^28^.

However, it is important to acknowledge the limitations of the model. First, the model was developed using retrospective data from a single vascular centre, which may restrict its generalizability to other settings and populations. Additionally, there is a possibility of unmeasured confounders that could impact the model’s accuracy. Furthermore, the measurement of compliance with medical therapy was not objectively assessed using appropriate tools, such as the eight-item Morisky Medication Adherence Scale (‘MMAS-8’)^34^, which may introduce potential bias into the results. Although missing data were appropriately handled, it is essential to recognize missing data as a limitation within the data set. Additionally, it is important to consider that the measurement of treatment compliance relied on self-declaration, which introduces the possibility of further confounding and potential inaccuracies in assessing true compliance levels. This potential self-declaration bias should be taken into account when interpreting the results and considering the limitations of the model. Furthermore, whilst the sample size of this study can be justified as appropriate for a proof-of-concept analysis, it is important to note that meticulous calculations or a larger sample size, ideally with at least 1000 events, would be necessary to further validate and establish the predictive accuracy of the model.

It is worth noting that the incidence of adverse outcomes observed in this study aligns with what has been reported in the existing literature, suggesting that the data set provides a reasonable representation of the target population. Moving forward, the next crucial step would be to validate this model in different settings and populations. By conducting external validation studies, the model’s performance and generalizability beyond the initial data set can be assessed, thereby strengthening its reliability and applicability in diverse clinical scenarios.

This study presents a novel ML model for predicting outcomes in patients with IC. It can provide personalized outcome forecasts for different management strategies, facilitating precise risk stratification and enhancing patient and clinician engagement. The model’s robustness, demonstrated through external validation, supports its potential for widespread application.

## Supplementary Material

zrad166_Supplementary_Data

## Data Availability

The data set and the algorithm are available from the corresponding author (B.R.) upon reasonable request.
