# Supplementary material for: Tailored risk assessment and forecasting in intermittent claudication
Source: BJS Open. 2024 Feb 27;8(1):zrad166. doi: 10.1093/bjsopen/zrad166 (PMC10898330; doi:10.1093/bjsopen/zrad166)
Supplement: zrad166_Supplementary_Data [file zrad166_supplementary_data.docx]

Tailored risk assessment and forecasting in intermittent claudication

Authors: Bharadhwaj Ravindhran^1, 3^,

Jonathon Prosser^1^

Arthur Lim ^1^

Bhupesh Mishra^2^

Ross Lathan^1^

Louise H Hitchman^1^

George E Smith^1^

Daniel Carradice^1^

Senior authors:

Ian C Chetter^1^

Dhaval Thakker^2^

Sean Pymer^1^

^1^ Academic Vascular Surgical Unit, 2^nd^ Floor, Allam diabetes centre, Hull Royal Infirmary, HU32JZ

^2^ School of Computer Science, University of Hull, Hull, UK

^3^ Department of Health Sciences, University of York, UK

Corresponding author: Bharadhwaj Ravindhran

Academic Vascular Surgical Unit

2^nd^ Floor, Allam diabetes centre

Hull Royal Infirmary

Hull HU32JZ

[Br965@york.ac.uk](mailto:Br965@york.ac.uk)

Orcid: 0000-0003-0778-2191

Twitter: bharathtiger

**Supplementary Materials - Index**

| **Supplementary Appendixes** |  |
| --- | --- |
| Supplementary Appendix 1:  Clinical characteristics included in the model  **Supplementary Results**  Supplementary Table 1: Baseline characteristics | *Page 3*  *Page 5* |

**Supplementary Appendix 1**

Clinical characteristics included in the model

| Sl | Clinical characteristics | Details |
| --- | --- | --- |
|  | Age | 18 and older |
|  | Sex | Male/Female  Non binary data unavailable |
|  | Diabetes | No diabetes  Type 1 Diabetes  Type 2 Diabetes |
|  | Hypertension | No hypertension  Primary hypertension ( Essential hypertension)  Secondary hypertension ( renovascular/endocrine causes) |
|  | Hyperlipidemia  ( total cholesterol >5.5mmol/L) | Presence or absence |
|  | Ischaemic heart disease | Presence or absence |
|  | Atrial fibrillation | Presence or absence |
|  | Cerebrovascular disease | Presence or absence |
|  | Previous percutaneous cardiac intervention or coronary artery bypass graft | Presence or absence |
|  | Chronic obstructive pulmonary disease | Presence or absence |
|  | Bronchial Asthma | Presence or absence |
|  | Active or concurrent malignancy | Presence or absence  Previous malignancy( treated/remission) |
|  | Body Mass Index | Categories:  0-18  18.1 -23.9  24 -30  >30.1 |
|  | Serum Albumin levels(g/l) | During the six months preceding or following the initial clinic visit. |
|  | Serum haemoglobin(g/l) | During the six months preceding or following the initial clinic visit. |
|  | Serum creatinine(μmol/L) | During the six months preceding or following the initial clinic visit. |
|  | Neutrophil-lymphocyte ratio | During the six months preceding or following the initial clinic visit. |
|  | Platelet-lymphocyte ratio | During the six months preceding or following the initial clinic visit. |
|  | Smoking status | Non smoker  Ex-Smoker  Current smoker |
|  | Duration of smoking | Less than 10 pack years  10-20 pack years  Greater than 20 pack years |
|  | Compliance to smoking cessation | Yes  No |
|  | Compliance to antiplatelet medication | Yes  No |
|  | Compliance to Statins/equivalent | Yes  No |
|  | Compliance to anticoagulation | Yes  No |
|  | Resting ankle-pressure brachial index | At first presentation |
|  | Self-reported claudication distance | At first presentation |
|  | Index leg | Unilateral or Bilateral |
|  | Aorto-iliac disease | Presence/absence  Unilateral/bilateral |
|  | Femoro-popliteal disease | Presence/absence  Unilateral/bilateral |
|  | Crural disease | Presence/absence  Unilateral/bilateral |

Supplementary table 1: Baseline characteristics of the training and testing cohorts.

| Characteristic | Training cohort  (n=255) | Testing cohort  (n = 254) | *p* value |
| --- | --- | --- | --- |
| Age, in years ( Mean/SD) | 68.8 ± 9.6 | 68.8 ± 9.2 | 0.895 |
| Male | 67.4% (n =172) | 68.9 % ( n=175) | 0.716 |
| Diabetes | 29.4(% n=75) | 35 % ( n= 89) | 0.176 |
| Hypertension | 59.6% (n=152) | 55.5 % ( n = 141) | 0.349 |
| Hyperlipidemia | 39.2% ( n = 100) | 37.0 % ( n = 94) | 0.609 |
| Ischaemic heart disease | 51.7% (n = 132) | 52.0 % (n = 133) | 0.946 |
| Atrial fibrillation | 19.6% ( n = 50) | 20.1 % ( n = 51) | 0.8877 |
| Cerebrovascular disease | 21.5% ( n = 55) | 25.6 % ( n = 65) | 0.276 |
| Previous PCI or CABG | 27.0% ( n = 69) | 26.4 % ( n = 67) | 0.878 |
| Serum Albumin levels(g/l)  (Mean/SD) | 36.9 ± 3.9 | 36.9 ± 3.9 | 0.930 |
| Serum haemoglobin(g/l)  (Mean/SD) | 134.8 ± 17.8 | 134.8 ± 18.6 | 0.071 |
| Serum creatinine(μmol/L)  (Mean/SD) | 93.5 ± 31.2 | 92.7 ± 32.8 | 0.786 |
| Neutrophil-lymphocyte ratio  (Mean/SD) | 3.4 ± 1.9 | 3.7 ± 2.3 | 0.067 |
| Platelet-lymphocyte ratio  (Mean/SD) | 175.2 ± 133.2 | 184.9 ± 137.9 | 0.419 |
| Non-smokers | 7.0% ( n = 18) | 9.4 % ( n = 24) | 0.335 |
| Smoking, pack years  <10 pack years  11-20 pack years  21-30 pack years  >30 pack years | 12.5 % ( n = 32)  36.0 % ( n = 92)  20.3 % ( n = 52)  30.9 % ( n = 79) | 12.2 % ( n = 31)  37.0 % ( n = 94)  20.5 % ( n = 55)  29.1 % ( n = 74) | 0.918  0.814  0.955  0.658 |
| Compliance to smoking cessation | 34.9 % ( n = 89) | 33.9% ( n = 86) | 0.812 |
| Compliance to antiplatelet medication | 63.5% ( n = 162) | 63 %( n = 160) | 0.907 |
| Compliance to Statin | 58.4% ( n = 149) | 54.3 % ( n = 138) | 0.351 |
| Resting ABPI  ( Mean/SD) | Right: 0.71 ± 0.2  Left: 0.74 ± 0.2 | Right: 0.69 ± 0.2  Left: 0.74 ± 0.2 | 0.305  1.000 |
| Self-reported claudication distance  Metres ( Mean/SD) | 137.7 ± 63.5 | 138.2 ± 63.2 | 0.92 |
| Aorto-iliac disease  Unilateral  bilateral | 29.8 % ( n = 76)  47.0 % ( n = 120) | 26.4 % ( n = 67)  42.9 %( n = 109) | 0.394  0.353 |
| Femoro-popliteal disease  Unilateral  bilateral | 39.2 %( n = 100)  50.9 % ( n = 130) | 37.4 %( n = 95)  52.4 %( n = 133) | 0.676  0.735 |
| Infra-popliteal disease  Unilateral  Bilateral | 29.0 %( n = 74)  18.0 %( n = 46) | 29.5 %( n = 75)  19.3 %( n = 49) | 0.901  0.706 |
| Initial treatment strategy:  SET  SET & EI  EI  Pharmacotherapy and exercise advice | 15.3% ( n = 39)  14.9% ( n = 38)  25.9% ( n = 66)  43.9% ( n = 112 ) | 13.8% ( n = 35)  13.0% ( n = 33)  31.5% ( n = 80)  41.7% ( n = 106) | 0.632  0.537  0.163  0.616 |

Legend: SD: standard deviation; PCI: percutaneous coronary intervention, CABG: coronary artery bypass graft, ABPI: Ankle-brachial pressure index, SET: supervised exercise therapy, EI: endovascular interventions
